# The Effect of *ACTN3* and *VDR* Polymorphisms on Skeletal Muscle Performance in Axial Spondyloarthropathies

**DOI:** 10.3389/fgene.2021.688984

**Published:** 2021-08-11

**Authors:** Isabel Pimenta, Hugo Mateus, Santiago Rodrigues-Manica, Rita Pinheiro-Torres, Agna Neto, Lúcia Domingues, Carolina Lage Crespo, Atlas Sardoo, Pedro Machado, Jaime C. Branco, Susana N. Silva, Fernando M. Pimentel-Santos

**Affiliations:** ^1^Chronic Diseases Research Center (CEDOC), NOVA Medical School, Universidade Nova de Lisboa, Lisboa, Portugal; ^2^Faculdade de Ciências, Universidade de Lisboa, Lisboa, Portugal; ^3^Faculdade de Ciências e Tecnologia, Universidade Nova de Lisboa, Lisboa, Portugal; ^4^Centro Hospitalar de Lisboa Ocidental, Hospital de Egas Moniz, Serviço de Reumatologia, Lisboa, Portugal; ^5^Instituto Politécnico de Setúbal, Escola Superior de Saúde, Setúbal, Portugal; ^6^Instituto de Higiene e Medicina Tropical, Universidade Nova de Lisboa, Lisboa, Portugal; ^7^Centre for Rheumatology and Department of Neuromuscular Diseases, University College London, London, United Kingdom; ^8^Center for Toxicogenomics and Human Health (ToxOmics), NOVA Medical School, Universidade Nova de Lisboa, Lisboa, Portugal

**Keywords:** spondyloarthropathies, muscle, muscle performance, *ACTN3*, *VDR*

## Abstract

**Background:**

Spondyloarthritis (SpA) are the most common group of chronic inflammatory rheumatic diseases affecting about 1.5% of the adult Caucasian population. Low back pain is the most common symptom. The aetiopathogenesis of SpA is multifactorial, with well-known genetic and environmental contributions. Furthermore, muscle properties might also be involved in the pathophysiological process and these could be modulated by the genetic background. *Alpha-actinin-3* (*ACTN3*) and *Vitamin D receptor* (*VDR*) genes are well-known genes related with muscle performance. Our aim was to analyze four SNPs of these genes and to evaluate their influence in axial SpA (axSpA) susceptibility, phenotype and muscle properties.

**Methods:**

We performed a pilot study based on case-control approach involving 56 participants: 28 axSpA patients and 28 healthy controls matched by age, gender and levels of physical activity. Clinical, epidemiological and muscle characterization data—muscle physical properties (stiffness, tone, and elasticity), strength, mass, and performance, were collected. Two different muscles were considered for analysis, the Multifidus and Gastrocnemius. Four SNPs of *ACTN3* (rs1815739) and *VDR* (rs2228570, rs731236, and rs7975232), were selected, analyzed and correlated with clinical, epidemiological and muscle characterization data.

**Results:**

In total, 51 individuals (27 axSpA patients and 24 matched controls) were eligible for further genetic analysis, 66.7% being male and with a mean age of 36 years. Muscle physical properties, muscle strength and muscle mass were similar in both groups; however, axSpA patients showed a decrease in muscle performance. None of the studied SNPs were associated with disease susceptibility/phenotype, muscle physical properties, muscle strength or muscle mass. However, *ACTN3* rs1815739 and *VDR* rs2228570 were shown to be associated with muscle performance.

**Conclusion:**

Our results suggest an association between *ACTN3* and *VDR* polymorphisms and muscle performance in axSpA.

## Introduction

Spondyloarthritis (SpA) is one of the most common groups of chronic inflammatory rheumatic diseases, affecting about 1.5% of the Caucasian adult population (Apostolakos et al., 2014; Costantino et al., 2018). SpA is typically characterized by inflammation of the spine and sacroiliac joints accompanied by pain, stiffness and in late stages, by reduced mobility. The disease may also affect the peripheral joints, periarticular structures (enthesitis, dactylitis), and extra-articular systems (acute anterior uveitis, psoriasis, and inflammatory bowel diseases) (Pimentel-Santos et al., 2012; Stolwijk et al., 2016; Costantino et al., 2018).

Entheses play a critical role in SpA ethiopathogeny and in the normal function of the musculoskeletal system. This structure not only allows the transmission of muscle contractile forces into the skeletal attachment site, but also participates in the dissipation of force from tendon into bone (Apostolakos et al., 2014).

The entheses have a unique immune microenvironment that can be stimulated through the combination of several factors (such as genetic predisposition, mechanical stress in the joints, and microbiota immune activation), leading to prostaglandin E2 release and IL-23-IL17 axis activation (Schett et al., 2017). This phenomenon leads to an influx of innate immune cells, promoting chronic inflammation in the entheses, followed by mesenchymal tissues responses and osteogenesis (Benjamin and Mcgonagle, 2001; Schett et al., 2017). In spite of all this information the underlying pathophysiological mechanisms for axial spondyloarthritis (axSpA) susceptibility remain unknown (Asquith et al., 2014; Gill et al., 2015; Van Mechelen and Lories, 2016).

The strong genetic component associated with the presence of HLA-B27 has been validated in different populations (Rosenbaum and Davey, 2011; Rudwaleit et al., 2011; Pimentel-Santos et al., 2013; Costantino et al., 2018). On the other hand, several loci and haplotypes relevant to disease susceptibility were identified through “Genome Wide Association Studies” (GWAS) (Sieper et al., 2009; International Genetics of Ankylosing Spondylitis et al., 2013; Osgood and Knight, 2018). Finally, expression studies also allowed the identification of genes related to inflammation, cartilage, bone and muscle metabolism (Pimentel-Santos et al., 2011). However, these studies can only explain a small portion of disease genetic predisposition and phenotype (Costantino et al., 2018).

In recent years, a link between biomechanical stress and axSpA susceptibility and severity has been raised. In axSpA patients and in animal models of the disease, the occurrence of microtrauma related to physical activities seems to induce inflammation and osteoproliferation within the spine (Mcgonagle et al., 2001; Jacques et al., 2014; Ramiro et al., 2015). Another study demonstrated an increased stiffness of the axial muscles in patients with axSpA (Andonian et al., 2015). Conceptually, microtrauma induced by daily activities (or by the muscle itself), may play an essential role in disease susceptibility/severity. Moreover, many studies support the notion that the gut microbiota plays an important role in axSpA through alterations of intestinal permeability, stimulation of immune responses, and molecular mimicry (Asquith et al., 2014; Gill et al., 2015). Thus, it is reasonable to speculate that general axSpA susceptibility and progression may result from a combination of host genetics, microbiota and micro-trauma. Still, how these factors interact with each other remains largely unknown (Benjamin and Mcgonagle, 2001; Simone et al., 2018). Further insights into genetic factors influencing muscle properties in axSpA patients will help unveil these complex interactions.

*Human Alpha- actinin 3* (*ACTN3*) and *Vitamin D receptor* (*VDR*) genes are associated with physical fitness and/or performance and muscular efficiency (Ceglia, 2009; Rejnmark, 2011; Pickering and Kiely, 2017). ACTN3 protein is a fast-twitch-specific isoform uniquely expressed in type-II muscle fibers, having an important role in the generation of contractile forces at high speeds (Pickering and Kiely, 2017). The *VDR* gene exhibits different PCR-RFLP single-nucleotide polymorphisms (SNPs), being *Bsm*I, *Fok*I, *Apa*I, and *Taq*I the most studied, which are thought to be associated with higher VDR activity (Ceglia, 2009; Hamilton, 2010; Rejnmark, 2011) or with protein function, also changing cellular responses to therapies (Hunt et al., 2009).

Thus, the aim of the present study is to characterize the association between *ACTN3* and *VDR* SNPs with axSpA susceptibility or disease characteristics, namely disease activity, functional, and metrological assessments, and in particular, the association with muscle physical properties, muscle strength, mass, and performance.

## Materials and Methods

### Populations Characterization

This pilot study enrolled 56 Caucasian individuals, of which 28 unrelated patients. All patients were previously diagnosed with axSpA and fulfill the Assessment of Spondyloarthritis international Society (ASAS) axSpA classification criteria (Rudwaleit et al., 2009) and the 28 healthy controls were matched by gender, age and level of physical activity. Cases were recruited from a Rheumatology outpatient clinic.

Eligible axSpA participants were recruited according to the following inclusion criteria: (1) axSpA, meeting the ASAS classification criteria, and symptom duration under 10 years; (2) Age between 18 and 50 years old; (3) Non-steroidal anti-inflammatory drugs (NSAIDs) and/or corticosteroids (equivalent to ≤ 10 mg of prednisone), in stable doses ≥ 4 weeks before screening was allowed; (4) Ability to provide informed consent. The exclusion criteria were: (1) Body mass index (BMI) ≥ 35 kg/m^2^, (2) Previous exposure to synthetic disease-modifying anti-rheumatic drugs (DMARDs) or biological disease-modifying anti-rheumatic drugs (bDMARDs); (3) Current pregnancy or breastfeeding; (4) Infection that required hospital stay, intravenous antibiotic treatment in the previous 30 days or oral antibiotic treatment within 14 days before screening; (5) Neoplastic disease (except for successfully treated squamous or basal cell carcinoma); (6) Any non-treated conditions (e.g., diabetes mellitus, ischemic heart disease); (7) Intra or peri-articular and tendon sheaths injections within 28 days prior to screening; (8) Rachis ankylosis (with syndesmophytes in all levels from D12 to S1, on lateral spine radiograph).

The current study was submitted and approved by both ethical committees of NOVA Medical School-Faculdade de Ciências Médicas, Universidade NOVA de Lisboa, Portugal and Centro Hospitalar Lisboa Ocidental, Hospital de Egas Moniz, EPE, Lisboa, Portugal. The study was conducted in accordance with International Conference on Harmonization good clinical practices and the Declaration of Helsinki. Voluntary written informed consent was obtained from all subjects before starting study procedures.

### Clinical Protocol

All 56 participants were submitted to a standardized protocol for extensive epidemiologic and muscle characterization. Physical activity was assessed according to the International Physical Activity Questionnaire (IPAQ) (Craig et al., 2003). The axSpA patients were also clinically evaluated (including Bath Ankylosing Spondylitis Disease Activity Index (BASDAI), Bath Ankylosing Spondylitis Functional Index (BASFI), disease duration, therapy). Additionally, Bath Ankylosing Spondylitis Metrology Index (BASMI) and myofascial characterization were performed by a single investigator (FPS), using different approaches:

(1) Muscle physical properties (stiffness, tone, and elasticity), assessed by a non-invasive device, the MyotonPro^®^, focusing on axial/torso (multifidus and longissimus dorsi) and on peripheral/lower limbs (gastrocnemius) muscles. The participants were in a prone position and measurements were taken after a 10 min rest.
(2) Muscle strength, measured using the Lafayette Manual Muscle Testing System for torso extension and lower limb extension; global strength evaluated through 5 times Sit-to-Stand test (STS5) (Cruz-Jentoft et al., 2019).
(3) Muscle Mass, assessed by bioimpedance, using direct segmental 8-point multifrequency bioelectric impedance analysis (InBody770, InBody Co., Ltd., Seoul, South Korea).
(4) Muscle performance, assessed by 60 s Sit-to-Stand test (ST60) and Gait speed (Cruz-Jentoft et al., 2019), using a 3D full-body kinematic model (Kinetikos^®^ Coimbra, Portugal).

Due to the absence of already established reference values for some variables related to physical muscular characteristics (stiffness, decrement/inverse of elasticity, tone), we have defined categories (low, intermediate and high) for the whole population group (for details see Supplementary Material), without taking into account the differences between genders because of the reduced sample size. Clinical variables were categorized according to the following cut offs in “not active/active disease” (BASDAI < 4, BASDAI ≥ 4), “Low/high functional ability” (BASFI < 4, BASFI ≥ 4) and low/high reduction in spinal range of motion (BASMI < 3, BASMI ≥ 3).

### Genotyping

Genomic DNA from all participants was isolated from peripheral blood samples using PureLink^TM^ Genomic DNA Mini Kit (Invitrogen) according to the manufacturer’s protocol instructions for blood lysates.

All samples were screened and genotyped for *ACTN3* and *VDR* genes polymorphisms: R557X (rs1815739) and ApaI (rs7975232), FokI (rs2228570), TaqI (rs731236), respectively, that have previously been associated with muscle performance in both men and women.

All SNPs studied (Table 1) were previously selected considering the Minor Allele Frequency (MAF) above or equal to 5% for European Caucasian population (HapMap CEU). SNPs under study belong to several parts of the gene: regulatory region, coding region or non-coding region.

**TABLE 1:** Identification of all genetic variants included in the study.

Gene	dsSNP ID (rs)	SNP	Variant type	A.A residue	Context sequence [VIC/FAM]
*ACTN3*	rs1815739	**R577X**	Non-sense	R/STOP	CAAGGCAACACTGCCCGAGGCTGAC**[T/C]**GAGAGCGAGGTGCCATCATGGGCAT
*VDR*	rs2228570	***Fok*I**	Missense	K/R	GGAAGTGCTGGCCGCCATTGCCTCC**[A/G]**TCCCTGTAAGAACAGCAAGCAGGCC
*VDR*	rs731236	***Taq*I**	Silent	I/I	TGGACAGGCGGTCCTGGATGGCCTC**[A/G]**ATCAGCGCGGCGTCCTGCACCCCAG
*VDR*	rs7975232	***Apa*I**	Intron	–	AAGGCACAGGAGCTCTCAGCTGGGC**[A/C]**CCTCACTGCTCAATCCCACCACCCC

*The bold values refers to the nucleotide change and the correspondence with the correspondent fluorocrome.*

The genotyping analysis was performed by quantitative polymerase chain reaction (qPCR) carried out on a 96-well QS5 Real-Time PCR (RT-PCR) System (Applied Biosystems; Thermo Fisher Scientific, Inc., Waltham, MA, United States), following the manufacturer’s instructions with the use of the commercially available TaqMan^®^ SNP Genotyping Assays (Applied Biosystems) detailed in Table 1. To confirm genotyping and ensure accurate results, inconclusive samples were reanalyzed, and genotyping was repeated in 10–15% of randomly chosen samples, with 100% concordance.

### Statistical Analysis

Data analysis was performed using the Statistical Package for the Social Sciences for Windows 22.0 version (SPSS, Inc.). All genotypes were coded accordingly in order to proceed with the statistical analysis. The analysis of Hardy-Weinberg frequencies for all alleles present in patients’ populations was carried out using exact probability tests available using the SNPStat software^1^ (Sole et al., 2006).

Participants’ demographic, clinical, and biomechanical characteristics were described and compared between healthy individual and patients with axSpA, using Chi-Square (χ^2^) for discrete data and Wilcoxon-Mann-Whitney-test for non-parametric continuous data.

Since this is not a conclusive final study, but an exploratory one on the role of selected polymorphisms in the *ACTN3* and *VDR* genes, and the data to be obtained should be looked at as proof of concept, the Bonferroni adjustment was deemed as not necessary as it is too conservative.

Logistic regression was used to estimate the risk of each muscle property modification when associated with each genotype: risk estimates were calculated under the codominant model and expressed as crude odds ratios (OR) and corresponding 95% confidence intervals (CI). Association between SNPs and the quantitative variables BASDAI, BASFI, BASMI, stiffness, decrement/inverse of elasticity, tone and strength were tested by linear regression. Results were considered significant when the corresponding two-tailed *p*-values were < 0.05. The most common homozygous genotype was considered the reference classes for such calculations.

## Results

### Epidemiological and Clinical Characterization

Even though the study started with 56 enrolled participants, in equal number of controls and patients, due to missing data in some physical measures and in individuals’ polymorphisms identification, the total sample population ended up comprising 51 individuals (27 axSpA patients and 24 controls). The axSpA patients, 66.7% male with a mean age of 36 ± 7 years old and a mean of 7.0 ± 0.9 years of disease duration. From the total of patients, 80.8% were HLA-B27 positive, 18.5% had active disease (BASDAI ≥ 4) and 14.8% presented high functional impairment (BASFI > 4). The majority of patients did not exhibit reduction in mobility (88.9% had BASMI < 3). There was no difference between patients and controls in terms of age, gender, level of physical activity and body mass.

Baseline characteristics are shown in detail, in Table 2, where all data comparison between patients and controls can be found.

**TABLE 2:** Study population characterization. Cases group (*n* = 27) and Healthy control group (*n* = 24).

Characteristics	Cases, *n* (%)	Mean ± SD	Controls, *n* (%)	Mean ± SD	*p*-value*
**Age**					
0–29	3 (11.1)		4 (16.7)		
30–39	14 (51.9)	36.8 ± 7.1	10 (41.7)	36.8 ± 7.7	
40–50	10 (37.0)		10 (41.7)		0.728
**Gender**					
Male	18 (66.7)	–	15 (62.5)	–	
Female	9 (33.3)		9 (37.5)		0.756
**Physical Activity level**					
Low	22 (81.5)	–	21 (87.5)	–	
High	5 (18.5)		3 (12.5)		0.555
***HLA-B27***					
Positive	21 (80.8)				
Negative	5 (19.2)	–	–	–	
Not available	1				–
Body height (cm)	–	170.4 ± 7.4	–	171.1 ± 8.8	
Body weight (kg)	–	75.9 ± 12.7	–	71.8 ± 12.9	
**BMI**					
Low weight	1 (4.2)		1 (4.3)		
Normoponderal	11 (45.8)		13 (56.5)	24.5 ± 3.7	0.410
Overweight	7 (29.2)	26.2 ± 4.3	8 (34.8)		
Obesity	5 (20.8)		1 (4.3)		
Not available	3		1		
**BASDAI**					
<4	22 (81.5)	3.0 ± 2.0	–	–	–
≥4	5 (18.5)				
**BASFI**					
≤4	23 (85.2)	2.2 ± 2.7	–	–	–
>4	4 (14.8)				
**BASMI**					
<3	24 (88.9)	1.1 ± 1.3	–	–	–
≥3	3 (11.1)				

*p-value determined by χ^2^-test.

### Clinical Data—Muscle Characteristics Analysis

We recorded and analyzed the results obtained from lumbar paravertebral muscle (Multifidus muscle) physical properties, namely: Muscle tonus (M.F); Muscle decrement, i.e., the inverse of elasticity (M.D) and Muscle stiffness (M.S). The regression analysis was performed individually for each characteristic (crude analysis) (Table 3).

**TABLE 3:** Characterization of multifidus muscle physical properties, strength and mass.

Characteristics	Cases *n* (%)	Controls *n* (%)	*p*-value*	ORcrude (95% CI)
**Muscle physical properties**				
**Muscle tonus^#^**				
Low	6 (22.2)	9 (37.5)		1.000 (reference)
Intermediate	9 (33.3)	10 (41.7)	0.185	1.350 (0.343–5.315)
High	12 (44.4)	5 (20.8)		3.600 (0.829–15.628)
**Muscle decrement^#^**				
Low	6 (22.2)	9 (37.5)		1.000 (reference)
Intermediate	12 (44.4)	7 (29.2)	0.406	2.571 (0.640–10.338)
High	9 (33.3)	8 (33.3)		1.687 (0.414–6.878)
**Muscle stiffness^#^**				
Low	5 (18.5)	11 (45.8)		1.000 (reference)
Intermediate	12 (44.4)	6 (25.0)	0.099	4.400 (1.041–18.599)†
High	10 (37.0)	7 (29.2)		3.143 (0.751–13.159)
**Muscle strength**				
**ST5**				
Normal	25 (92.6)	22 (100.0)	0.192	N.D.
Reduced	2 (7.4)	0 (0.0)		
**Muscle mass**				
**Muscle mass**				
Low	1 (4.2)	22 (100.0)		
Normal range	19 (79.2)	0 (0.0)	0.724	N.D.
Above	4 (16.7)			
**Muscle performance**				
**ST60^#^**				
Low	9 (33.3)	0 (0.0)	**0.008**	N.D.
Intermedium	8 (29.6)	7 (31.8)		
High	10 (37.0)	15 (68.2)		
**Gait speed**				
Good	6 (27.3)	12 (57.1)	**0.047**	1.000 (reference)
Low	16 (72.7)	9 (42.9)		3.556 (0.993–12.733)^‡^

*p- value determined by χ^2^-test; ^#^Swiss reference values were used. ^‡^borderline effect (P-value = 0.051). BMI (Body mass Index); ST5 (5 Times Sit-to-Stand Test); ST60 (60 s Sit-to-Stand Test). ^#^Categorization parameters. ^†^*p*-value = 0.044. The bold values refers to values satistical significants.

No significant differences in muscle physical properties, muscle strength (ST5) and muscle mass were identified between patients and controls. However, it seems that patients tend to express higher levels of stiffness in multifidus muscle (trunk) (Table 3). Muscle performance, measured by ST60 and gait speed, was significantly reduced in patients, compared to controls (*p* < 0.05).

### Genotyping and Individual Susceptibility Analysis

After genotyping analysis, the SNPs distribution was performed and their genetic contribution to disease susceptibility was evaluated (Table 4). To perform the correlations, we deemed it important to verify if the four SNPs were in Hardy-Weinberg Equilibrium (HWE). All the populations followed the Hardy-Weinberg Equilibrium (HWE), except for *Taq*I SNP (*p*-value = 0.021).

**TABLE 4:** Genotype distribution between axSpA patients and controls, for *ACTN3* and *VDR* polymorphisms: rs1815739 (R577X); rs2228570 (*Fok*I), rs731236 (*Taq*I), and rs7975232 (*Apa*I).

Genotype	Cases *n* (%)	Controls *n* (%)	*p*-value*	OR^a^ (95% CI)
***ACTN3 R577X***				
C/C	8 (30.8)	6 (25.0)		1.000 (reference)
C/T	15 (57.7)	12 (50.0)	0.463	0.375 (0.066–2.145)
T/T	3 (11.5)	6 (25.0)		0.938 (0.255–3.449)
C/T + T/T	18 (69.2)	18 (75.0)		0.750 (0.216–2.602)
***VDR—Fok*I**				
A/A	4 (15.4)	2 (8.3)		1.000 (reference)
A/G	12 (46.2)	16 (66.7)	0.339	0.375 (0.059–2.397)
G/G	10 (38.5)	6 (25.0)		0.833 (0.115–6.013)
A/G + G/G	22 (84.6)	22 (91.7)		0.500 (0.083–3.017)
***VDR*—*Taq*I**				
A/A	11 (42.3)	7 (30.4)		1.000 (reference)
A/G	7 (26.9)	9 (39.1)	0.599	0.495 (0.126–1.945)
G/G	8 (30.8)	7 (30.4)		0.727 (0.181–2.914)
A/G + G/G	15 (57.7)	16 (69.6)		0.597 (0.183–1.943)
***VDR*—*Apa*I**				
C/C	7 (28.0)	3 (12.5)		1.000 (reference)
C/A	9 (36.0)	10 (41.7)	0.400	0.351 (0.070–1.761)
A/A	9 (36.0)	11 (45.8)		0.386 (0.076–1.959)
C/A + A/A	18 (72.0)	21 (87.5)		0.367 (0.083–1.633)

*p-value χ^2^-test. Abbreviations: a ORs and 95% CI for specific categories were calculated using logistic regression models; OR, odds ratio; CI, confidence interval.

Considering that three SNPs from the same gene (*VDR*) were analyzed, we also evaluated the possibility of establishing an haplotype. However, the allele combination obtained for our populations did not reveal a statistically relevant combination to be correlated with disease susceptibility. After a multiple-SNP analysis, the results showed the existence of *Linkage Disequilibrium* between *Apa*I and *Taq*I SNPs from *VDR* (D’ = 0.9382, *p*-value ≤ 0.001).

Another question we wanted to address was related to the potential role of *ACTN3* and *VDR* genes polymorphisms in the etiology of the disease evaluating the risk magnitude. The genotypic frequencies were determined for both groups and for all SNPs under study.

According to the results obtained, the studied SNPs did not seem to be associated with an increased risk of axSpA susceptibility.

In addition, axSpA clinical parameters (BASDAI, BASFI, and BASMI) were also measured in the patient population and the association with the allelic distribution for each SNP was investigated (Table 5). *ACTN3* and *VDR* polymorphisms did not markedly influence axSpA disease activity, physical function or severity, as measured by BASDAI, BASFI, and BASMI.

**TABLE 5:** Clinical characteristics across *ACTN3* and *VDR* genes polymorphisms.

SNPs	BASDAI			BASFI			BASMI			
	Cases *n* (%)		*p*-value*	Cases *n* (%)		*p*-value*	Cases *n* (%)		*p*-value*	
	No active disease	Active disease		Low functional repercussion	High functional repercussion		Mild score	Severe score		Controls *n* (%)
***ACTN3* R577X**										
C/C	6 (28.6)	2 (40.0)		6 (27.3)	2 (50.0)		8 (34.8)	0 (0.0)		6 (25.0)
C/T	12 (57.1)	3 (60.0)	0.700	13 (59.1)	2 (50.0)	0.633	12 (52.2)	3 (100.0)	0.398	12 (50.0)
T/T	3 (14.3)	0 (0.0)		3 (13.6)	0 (0.0)		3 (13.0)	0 (0.0)		6 (25.0)
***VDR—Fok*I**										
A/A	3 (14.3)	1 (20.0)		3 (13.6)	1 (25.0)		4 (17.4)	0 (0.0)		2 (8.3)
A/G	9 (42.9)	3 (60.0)	0.534	9 (40.9)	3 (75.0)	0.247	9 (39.1)	3 (100.0)	0.188	16 (66.7)
G/G	9 (42.9)	1 (20.0)		10 (45.5	0 (0.0)		10 (43.5)	0 (0.0)		6 (25.0)
***VDR—Taq*I**										
A/A	9 (42.9)	2 (40.0)		11 (50.0)	0 (0.0)		10 (43.5)	1 (33.3)		7 (30.4)
A/G	5 (23.8)	2 (40.0)	0.810	5 (22.7)	2 (50.0)	0.323	6 (26.1)	1 (33.3)	0.886	9 (39.1)
G/G	7 (33.3)	1 (20.0)		6 (27.3)	2 (50.0)		7 (30.4)	1 (33.3)		7 (30.4)
***VDR*—*Apa*I**										
C/C	6 (28.6)	1 (25.0)		7 (31.8)	0 (0.0)		6 (26.1)	1 (50.0)		3 (12.5)
C/A	7 (33.3)	2 (50.0)	0.692	8 (36.4)	1 (33.3)	0.415	9 (39.1)	0 (0.0)	0.528	10 (41.7)
A/A	8 (38.1)	1 (25.0)		7 (31.8)	2 (66.7)		8 (34.8)	1 (50.0)		11 (45.8)

*p-value χ^2^-test; BASDAI < 4, “no active disease”; BASDAI ≥ 4 “active disease”; BASFI < 4 “low functional repercussion”; BASFI ≥ 4 “high functional repercussion,” and BASMI < 3, low reduction, BASMI ≥ 3, high reduction in movement amplitude.

We hypothesize how relevant the genetic background might be to explain muscle properties (physical-stiffness, tone, elasticity; strength; mass) and in particular physical performance (ST60 and Gait Speed), where statistically significant differences between patients and controls were registered. To understand the hypothetical effect of single SNP in these parameters, we applied the logistic regression model adjusted to the presence of the genetic component; the results are presented in the table below (Table 6).

**TABLE 6:** Association of *ACTN3* and *VDR Fok*I polymorphisms and gait speed parameters.

Characteristics	ORcrude (95% CI)	ORadjusted to *ACTN3* (95% CI)	ORadjusted to *VRD*—Fok1 (95% CI)
**Gait speed**			
Good	1.000 (reference)	1.000 (reference)	1.000 (reference)
Low	3.556 (0.993–12.733)^a^	**3.911 (1.044–14.658)^b^**	**3.785 (1.025–13.982)^c^**

**ORs and 95% CI for specific categories were calculated using logistic regression models; OR, odds ratio; CI, confidence interval. ^*a*^p-value = 0.051; ^*b*^p-value = 0.043; ^*c*^p-value = 0.047.* The bold values refers to values satistical significants.*

Data shown refers only to *ACTN3* and *VDR* Fok1 polymorphisms, since the logistic regression of the other two SNPs did not show statistically significant results.

## Discussion

This pilot study, involving 27 young axSpA patients with short disease duration, has not shown any difference in muscle physical properties, global muscle strength or mass compared to controls. However, a reduction in muscle performance assessed by two different approaches, ST60 and Gait speed, was found. Even using a small participant’s sample, the overall results allowed us to generate relevant data on disease susceptibility evidencing low physical performance in spondiloarthritis patients. Thus, we considered interesting to examine if genes associated with muscle performance, such as *ACTN3* and *VDR*, might contribute to explain such differences.

As expected, due to our selection criteria, both groups (SpA patients and controls) are largely similar, regarding age, gender, levels of physical activity. When analyzing disease characteristics, the majority of patients exhibit low disease activity, low functional and low metrological repercussion. Most of our patients are HLA-B27 positive (80.8%), which is in line with the percentages already found for the Portuguese population (Pimentel-Santos et al., 2012). Due to the small size of our study population and low number of female individuals, we did not take into account possible gender differences in any of our analysis.

In this study, we selected several SNPs of well-known genes—*ACTN3* (Ma et al., 2013; Pratt et al., 2019) and *VDR* (Pratt et al., 2019)—related with muscle performance to evaluate their influence in axSpA susceptibility, axSpA phenotype and in muscle properties. In this context, we were interested in studying these genetic variants in the axSpA context looking for the variants related with low muscle performance and simultaneously evaluate any association with muscle physical properties, strength, and lean mass. This would represent an additional method to identify patients that might benefit from a program of physical exercise and to the identification of the best modalities to be used in clinical practice.

In our study, we demonstrate that *ACTN3* and *VDR* are not significantly associated with either susceptibility to axSpA or measures of its activity, disability or severity, as measured by BASDAI, BASFI, and BASMI, respectively. Indeed, no association has been identified in GWAS between these genes and SpA (Australo-Anglo-American Spondyloarthritis et al., 2010; International Genetics of Ankylosing Spondylitis et al., 2013). Nevertheless, some *VDR* polymorphisms have been linked to some musculoskeletal diseases, such as idiopathic scoliosis susceptibility or curve severity, herniation and spinal tissues degeneration and rheumatoid arthritis (Saad et al., 2015; Di Spigna et al., 2016; Vieira et al., 2018; Li et al., 2019).

No association was established between the studied SNPs and muscle physical properties, namely stiffness, tone or elasticity. To the best of our knowledge, this association was never studied. Again, no association for overall strength (ST5) was registered in our cohort. However, in several previous studies, *ACTN3* 577R allele and *VDR* were associated with higher levels of strength. The rs540874 polymorphism of *ACTN3* gene was associated with the muscle function of lower limb (women with the G allele were likely to have higher strength compared with the ones with A allele) but not with the higher limb, in postmenopausal women. Interestingly, in the same study, the improvement of muscle strength after an intervention (exercise and Vitamin D supplementation) was possibly correlated with rs540874, rs618838, and rs2229456 polymorphisms (Xue et al., 2018). A significant association between *VDR* genotype and quadriceps (23% difference) and grip (7% difference) strength was observed in non-obese elderly women (Geusens et al., 1997).

The analysis of these genetic markers regarding lean muscle mass did not show, again, any difference between both groups. It is well-known that genetic factors account for approximately 50-80% of inter-individual variation in lean body mass, with impacts detected on both “training-naïve” muscle mass and its growth response (Puthucheary et al., 2011). Indeed, these genes have been found to contribute to variation in lean body mass and bone mass density, contributing to understanding the molecular bases of sarcopenia and osteoporosis (Tan et al., 2012; Gonzalez-Mercado et al., 2013; Cho et al., 2017; Scimeca et al., 2018). However, in one study involving older Caucasian men, whole body and thigh non-skeletal lean mass were independent of *ACTN3* R/X polymorphisms (Mccauley et al., 2010). In contrast, VDR expression decreases with age and *VDR* genotype seems to be associated with fat-free mass in elderly men and women (Puthucheary et al., 2011). Differences in populations’ characteristics, study methodologies and reduced number of participants (underpowered studies) to detect genes with small effects, potentially lead to discrepancies between results.

This study has shown a clear reduction in axSpA muscle performance without changes in muscle physical properties, strength, and mass. This observation allows us to speculate about a possible muscle dysfunction. As genetics has a strong influence in overall axSpA susceptibility, it is of main interest to investigate a possible genetic base to explain this impairment in muscle performance. Our results indicate that *ACTN3* R577X and *VDR* Fok1 SNPs might influence Gait Speed [OR, 3.911; 95% CI (1.044–14.658) and OR, 3.785; 95% CI (1.025–13.982)]. Several studies have shown an association of these variants on muscle performance. In 2003, Yang and colleagues (Yang et al., 2003) demonstrated a significant association between *ACTN3* genotype and athletic performance. They found that both male and female elite sprint athletes have significantly higher frequencies of the 577R allele compared to controls. Later on, several papers consistently reported a strong association between RR genotype and elite power performance (Paparini et al., 2007; Papadimitriou et al., 2008; Eynon et al., 2009; Chiu et al., 2011; Ma et al., 2013). Similar evidence was documented for *VDR* polymorphisms (Micheli et al., 2011; Puthucheary et al., 2011). In elite Italian soccer players, an interaction of two polymorphisms (*ACE* and *ACTN3*) predicted explosive leg-muscle strength, however, the contribution of genetic factors was only 23.92% (Massidda et al., 2012).

The genotype distribution of all SNPs was in HWE, except for the *Taq*I polymorphism (*p* = 0.021), suggesting the influence of genetic drift due to the small population size. Furthermore, we also evaluated the possibility to establish a specific haplotype for *VDR* gene polymorphisms, however, no haplotype was identified as relevant. Our results allowed us to identify *Linkage Disequilibrium* between *Apa*I and *Taq*I SNPs, indicating that both are segregated together. This corroborates some studies (Hamilton, 2010) and means that both SNPs are always transmitted in block (Cieslinska et al., 2018) (data not shown), even though it was not possible to identify a risk haplotype for our population. Despite the literature revealing that *VDR* SNPs constitute a haplotype, due to their proximity, we could not confirm those reports (Hunt et al., 2009).

To our knowledge, this pilot study is the very first including a genetic susceptibility analysis for muscle properties in the axSpA context. Overall, our results suggest an association between *ACTN3* and *VDR* polymorphisms and muscle performance in the axSpA context. This opens the door to a better stratification of patients regarding exercise programs, but also to identify other candidate genes that might help to characterize the genetic background of the disease.

## Data Availability Statement

The raw data supporting the conclusions of this article will be made available by the authors, without undue reservation.

## Ethics Statement

The studies involving human participants were reviewed and approved by the NOVA Medical School | Faculdade de Ciências Médicas, Universidade NOVA de Lisboa, Portugal Centro Hospitalar Lisboa Ocidental, Hospital de Egas Moniz, EPE, Lisboa, Portugal. The patients/participants provided their written informed consent to participate in this study.

## Author Contributions

FP-S, JB, and SS mainly developed the conceptualization. IP, HM, FP-S, and SS performed the methodology. SS and FP-S proceeded validation and prepared the visualization. SS and IP did formal analysis. FP-S, SR-M, RP-T, AN, LD, CL, IP, HM, and SS mainly performed the investigation. FP-S collaborated resources acquired in restrict. AN, FP-S, and SS performed the data curation. FP-S, SS, and IP contributed to the writing – original draft preparation. FP-S, SS, SR-M, RP-T, AN, LD, CL, AS, PM, and JB contributed to the writing – review and editing. FP-S, JB, and SS supervised this project. LD contributed to the project administration. FP-S and JB contributed to the funding acquisition. All authors contributed to the article and approved the submitted version.

## Conflict of Interest

The authors declare that the research was conducted in the absence of any commercial or financial relationships that could be construed as a potential conflict of interest.

## Publisher’s Note

All claims expressed in this article are solely those of the authors and do not necessarily represent those of their affiliated organizations, or those of the publisher, the editors and the reviewers. Any product that may be evaluated in this article, or claim that may be made by its manufacturer, is not guaranteed or endorsed by the publisher.

## Footnotes

1
http://bioinfo.iconcologia.net/SNPstats
