# Supplementary material for: The Effect of ACTN3 and VDR Polymorphisms on Skeletal Muscle Performance in Axial Spondyloarthropathies
Source: Front Genet. 2021 Aug 11;12:688984. doi: 10.3389/fgene.2021.688984 (PMC8385750; doi:10.3389/fgene.2021.688984)
Supplement: Supplementary file 1 [file Data_Sheet_1.docx]

# Supplementary Material

**Multifidus muscle evaluation**

For the following muscle parameters: Muscle tonus, Muscle Decrement Muscle Stiffness, cut off was calculated using only reference values of HC and terciles (P33-P66). It is important to highlight that although it is known that tonus and rigidity exhibit differences between both genders (male and female), due to the small size of our sample, this fact was no taken into account for the cut off values definition and analysis.

**Muscle tonus – Cut-Offs**

| 0- 15.60 | Low tonicity |
| --- | --- |
| 15.61- 16.80 | Intermedium |
| ≥ 16.81 | High tonicity |

**Muscle Decrement – Cut-Offs**

| 0-1.16 | High elasticity |
| --- | --- |
| 1.17- 1.36 | Intermedium |
| ≥1.37 | Low elasticity |

**Muscle Stiffness – Cut-Offs**

| 0-280.50 | Low stiffness |
| --- | --- |
| 281-311 | Intermedium |
| ≥ 312 | High stiffness |
